# On the Relationship between Multicast/Broadcast Throughput and Resource Utilizations in Wireless Mesh Networks

**DOI:** 10.1155/2013/794549

**Published:** 2013-11-19

**Authors:** Avid Avokh, Ghasem Mirjalily, Jamshid Abouei, Shahrokh Valaee

**Affiliations:** ^1^Faculty of Electrical and Computer Engineering, Yazd University, Yazd 8915818411, Iran; ^2^Department of Electrical and Computer Engineering, University of Toronto, Toronto, ON, Canada M5S 3G4

## Abstract

This paper deals with the problem of multicast/broadcast throughput in multi-channel multi-radio wireless mesh networks that suffer from the resource constraints. We provide a formulation to capture the utilization of the network resources and derive analytical relationships for the network's throughput in terms of the node utilization, the channel utilization, and the number of transmissions. Our model relies on the on-demand quality of service multicast/broadcast sessions, where each admitted session creates a unique tree with a specific bandwidth. As an advantage, the derived relationships are independent of the type of tree built for each session and can be used for different protocols. The proposed formulation considers the channel assignment strategy and reflects both the wireless broadcast advantage and the interference constraint. We also offer a comprehensive discussion to evaluate the effects of load-balancing and number of transmissions on the network's throughput. Numerical results confirm the accuracy of the presented analysis.

## 1. Introduction

Wireless mesh networks (WMNs) have been recognized as a new class of multihop networks that provide low-cost solutions for broadband wireless applications [1]. A WMN is composed of three types of nodes: gateways, mesh routers, and mesh clients [1, 2]. Gateways enable the integration of various networks, for example, Wi-Fi, Zigbee, WiMAX, and cellular networks. Mesh routers have minimal mobility and form the backbone of the network. They have the functionality of both an access point and a relay node. As a relay node, mesh routers forward the packets from the source node to the destination nodes. However, as an access point, they provide network access for mesh clients within their coverage area. One challenge in WMNs is the degradation of the network's capacity due to the co-channel interference. This problem has motivated the researchers to improve the network's throughput using efficient schemes. An effective approach to mitigate the co-channel interference is to equip the mesh routers with multiple radios tuned to non-overlapping channels. The ability to utilize multiple radios allows the mesh routers to send/receive packets simultaneously on distinct channels and, therefore, increases the bandwidth available to the network [2]. However, due to the limited number of radios and non-overlapping channels, some links interfere with each other and cannot be active at the same time. These resource constraints degrade the performance of Multi-Channel Multi-Radio WMNs (MCMR-WMNs). Thus, a proper resource assignment strategy is required to improve the performance of such networks.

On the other hand, recently, the popularity of multimedia services such as IP-TV, video conference, and distant education, has significantly increased [3–5]. In this regard, the multicast routing provides underlying facilities for the multimedia applications in WMNs. The basic difference between multicast routing in wireless and wired networks is the broadcast nature of the wireless medium that results in a well-known property named wireless broadcast advantage (WBA) [6, 7]. Based on the WBA, a single transmission in a node simultaneously covers multiple neighboring receivers. Minimizing the number of transmissions improves the utilization of the network resources and subsequently increases the network's throughput. Besides the number of transmissions, the load-balancing problem is another pertinent issue to be considered in MCMR-WMNs. This problem can be discussed from two perspectives: spatial load-balancing and channel load-balancing. From the viewpoint of a specific channel, when a part of the network experiences congestion, the new traffic flows should not be routed through that part. In addition, from the perspective of a specific location, the traffic load must be balanced over all available channels in the network. If the traffic load in the network is balanced, the interference will be decreased, and more resources will be available for accepting the future traffics.

To evaluate the performance of a MCMR-WMN, there are several criteria that often interact with each other. Thus, it is not possible to draw a clear boundary between them. In this regard, different approaches try to improve different aspects of the networks. One difficulty in comparing different schemes is the lack of a standard benchmark. Certainly, having a prior knowledge about the bounds of criteria and their corresponding relationship provides more options for the system administrator to control the parameters of the network.

Taking the above challenges into account, the main goal of this paper is to quantify the throughput in MCMR-WMNs. We focus on the scenario of multicast and broadcast sessions, where each session has a specific bandwidth requirement. On-demand requests arrive dynamically one by one without any prior knowledge of future arrivals. A session will be accepted if a routing tree with sufficient bandwidth on each link can be established. In particular, we provide a formulation to express the network's throughput in terms of the node utilization, the channel utilization, and the number of transmissions. We also discuss how appropriate use of the resources affects the network's throughput.

The rest of the paper is organized as follows.  Section 2 surveys the previous related works. The details of the network model are described in Section 3.  Section 4 presents a formulation to capture the resource utilizations. In Section 5, we derive analytical expressions for the multicast/broadcast throughput in both small-scale MCMR-WMN and large-scale MCMR-WMN.  Section 6 presents the numerical results and the discussion. Finally, some concluding remarks are provided in Section 7.

## 2. Related Work

 In past years, different aspects of MCMR-WMNs have been widely studied for unicast flows. In particular, some works endeavor to heuristically improve the network's performance [2, 8], while others focus on the optimization solutions [9–11]. In this regard, several routing metrics (such as ETX, ETT, WCETT, WCCETT, MIC, iAWARE, and MIND [1, 12]) have been proposed. However, due to the differences between unicast and multicast routing, the designed unicast schemes cannot be efficient for the multicast traffics. In one of the most fundamental researches on the multicast routing, the work in [4] compares the performance of the Minimum Steiner Trees (MSTs) and the Shortest Path Trees (SPTs) in Single-Channel Single-Radio WMNs (SCSR-WMNs). Experimental results in [4] show that SPTs offer a better performance than MSTs. In addition, the implementation difficulty of MSTs is another factor that makes SPTs more favorable in WMNs. However, none of these protocols consider the problems of load-balancing and resource utilization.

Roy et al. [13] study the high-throughput metrics for multicast routing in WMNs. They point out the difference between unicast and multicast routing and show how to adapt the unicast routing metrics for use in multicast flows. They also propose a low-overhead adaptive algorithm to incorporate the link-quality-based metrics to a representative multicast routing protocol.

As mentioned before, the ability to make an appropriate use of the network resources can efficiently increase the throughput in WMNs. In line with this concept, some works aim to minimize the number of transmissions required to deliver one packet from the source to all the destinations [14–16]. The authors in [15] propose a multicast routing metric, named Multi-Channel Minimum Number of Transmissions (MCMNT), that considers the wireless broadcast advantage and the channel diversity to minimize the network bandwidth consumed by the routing tree. MCMNT also tries to minimize the intra-flow interference in the network. Zeng et al. [16] propose two algorithms, named Level Channel Assignment (LCA) and Multi-Channel Multicast (MCM), to minimize the number of forwarding nodes and the total hop-count distances in MCMR-WMNs. These algorithms reduce the intra-flow interference using the heuristic channel assignment strategies. The authors in [16] show that the LCA and the MCM algorithms outperform the single-channel multicast in terms of the throughput and the delay. They also demonstrate that using all the partially overlapping channels instead of only the non-overlapping channels can further diminish the interference in the network.

In [17], Chiu et al. challenge the load-balancing issue in MCMR-WMNs. The basic idea in [17] is that if the traffic on the most-heavily loaded channel is minimized, the traffic load in the network will be balanced. In this way, they first present an integer linear programming formulation to optimally construct the bandwidth-guaranteed broadcast trees. Then, an efficient algorithm is proposed to heuristically improve the call acceptance rate in the network. Reference [18] proposes two load-aware metrics, named “Flow Load Multicast Metric (FLMM)” and “Reliable Flow Load Multicast Metric (FLMM^R^)”, for multicast routing in MCMR-WMNs. Although both metrics count the interference and the WBA, the latter case further considers the unreliability of the IEEE 802.11 MAC protocol. FLMM^R^ uses the Packet Delivery Ratio (PDR) of the wireless links and reduces retransmission overheads. In line with this concept, the work in [19] suggests two distributed strategies, named “Multicast Auto-Rate Selection (MARS)” and “MARS-Retransmit (MARS-R)”. The MARS scheme uses PDR of the wireless links at various transmission rates. The MARS-R algorithm facilitates the joint use of rate control and link-layer mechanisms (such as acknowledgments and retransmissions) to improve the reliability of high-throughput multicast flows.

During recent years, we have considered the problem of traffic engineering for multicast/broadcast flows in WMNs [3, 7, 20]. The work in [3] studies the special case of broadcasting for the small-scale WMNs. In [7], we present an Interference-Aware Joint Channel and Rate Selection (IA-JCRS) algorithm to choose the best transmission rates and the best transmission channels for a given fixed routing tree. However, being bound to a routing tree reduces the freedom to choose the alternative feasible paths. Indeed, using a joint interference-aware routing scheme leads to a better utilization of the network resources. Accordingly, in [20], we propose two cross-layer algorithms, named the “Interference- and Rate-aware Multicast Tree (IRMT)” and the “Interference- and Rate-aware Broadcast Tree (IRBT).” As an advantage, the proposed algorithms jointly address the problems of multicast/broadcast routing tree construction, transmission rate selection, transmission channel selection, and call admission control.

One drawback of the previous works is that they pay less attention to the theoretical analysis of multicast/broadcast flows. Most of the literature tries to propose some heuristically or optimally solutions to improve different aspects of the network. Unlike the previous works, this paper quantifies the multicast and the broadcast throughput in both small-scale MCMR-WMN and large-scale MCMR-WMN. In this regard, we also present a simulation-based discussion to simultaneously study the effects of load-balancing and number of transmissions on the network's throughput.

## 3. Network Model and Assumptions 

 We consider a typical MCMR-WMN consisting of *n* stationary nodes (In this paper, the terms “mesh router” and “node” are used interchangeably for convenience.). Each node *x* is equipped with *R*_*x*_ half-duplex radios tuned to one of the *K* available non-overlapping channels where no channel switching is allowed. For the sake of efficiency, the radios of a node are tuned to different non-overlapping channels. When a radio of a node transmits or receives the packets on a channel, other radios of the same node are able to communicate at the same time with neighboring nodes on other channels. In this paper, a single-rate framework is assumed for all link-layer transmissions. In addition, we suppose that the radios of the nodes are equipped with omnidirectional antennas characterized by the same transmission range and the same interference range. Node *x* is directly connected to node *y* and forms a wireless link, if and only if node *y* is within the transmission range of node *x* and they share a common channel. In this regard, we model the network as a directed graph **G** = (**V**, **E**), where **V** = {*v*_1_, *v*_2_,…, *v*_*n*_} is the set of vertices representing *n* nodes and **E** denotes the set of communication links. In this work, we consider the traffic model of on-demand multicast/broadcast sessions, where each admitted session creates a unique tree with a specific bandwidth requirement. In this way, we adopt a schedule-based MAC protocol, in which the conflict-free transmission is ensured by assigning the interfering transmitters to either send on different non-overlapping channels or send on the same channel but at different time slots.

## 4. Problem Formulation 

 The limited number of radios and the shared nature of wireless medium impose some resource constraints on MCMR-WMNs. In this section, we derive a formulation to capture the utilization of the network resources and to analyze the feasibility of multicast/broadcast session requests.

**Definition 1**

The capacity of the *i*th mesh router is defined as *C*(*i*) = *R*_*i*_ *c*_0_, where *R*_*i*_ represents the number of its radios and *c*_0_ is the capacity of the channels. In addition, we define the sent and the received traffic loads of the *i*th mesh router, denoted by *l*_*s*_(*i*) and *l*_*r*_(*i*), respectively, as *l*_*s*_(*i*) = ∑_*b*=1_^*R*_*i*_^ *l*_*s*_*b*__(*i*) and *l*_*r*_(*i*) = ∑_*b*=1_^*R*_*i*_^ *l*_*r*_*b*__(*i*), *i* = 1,…, *n*, where *l*_*s*_*b*__(*i*) and *l*_*r*_*b*__(*i*) are the sent and the received traffic loads of the *b*th radio in the *i*th mesh router, respectively. Thus, the total traffic load of the *i*th mesh router is obtained as *l*(*i*) = *l*_*s*_(*i*) + *l*_*r*_(*i*), *i* = 1,…, *n*.

According to the assumption of the half-duplex radios, each radio can only send or receive on a fixed channel *k* at any time slot; therefore, it is required that

(1) $$\begin{array}{c} l_{s_{b}}\left(i\right)+l_{r_{b}}\left(i\right)\leq c_{0},\;\forall i\in V,\;\;b=1,\ldots ,R_{i}. \end{array}$$

Let *l*_*i*_^*j*^ denote the created load by the *j*th session on the *i*th mesh router. Thus, the total load of the *i*th mesh router can be rewritten as *l*(*i*) = ∑_*j*=1_^*n*_*s*_^ *l*_*i*_^*j*^, *i* = 1,…, *n*, where *n*_*s*_ is the number of active sessions. In general, each multicast/broadcast tree *T* ^*j*^ is composed of a set of MAC multicast transmissions on different nodes and channels. The number of transmissions of node *i* at the *j*th tree, denoted by NT_*i*_^*j*^, is given by

(2) $$\begin{array}{c} \text{N}{\text{T}}_{i}^{j}=\sum_{k\in K}q_{i,k}^{j},\;i=1,\ldots ,n, \end{array}$$

where **K** is the set of *K* available non-overlapping channels and *q*_*i*,*k*_^*j*^ = 1 if node *i* is a forwarding node on channel *k* at the *j*th tree, and *q*_*i*,*k*_^*j*^ = 0 otherwise. In this case, we define the total number of transmissions for the *j*th tree as

(3) $$\begin{array}{c} \text{NT}\left(T^{j}\right)=\sum_{i\in V}\text{N}{\text{T}}_{i}^{j}. \end{array}$$

In fact, NT(*T* ^*j*^) shows the number of transmissions required to deliver one packet from the source node to all the destinations at the *j*th multicast/broadcast tree. Thus, the average number of transmissions per active session is expressed as

(4) $$\begin{array}{c} \bar{\text{NT}}=\frac{1}{n_{s}}\sum_{j=1}^{n_{s}}\text{NT}\left(T^{j}\right). \end{array}$$

**Figure 1:**
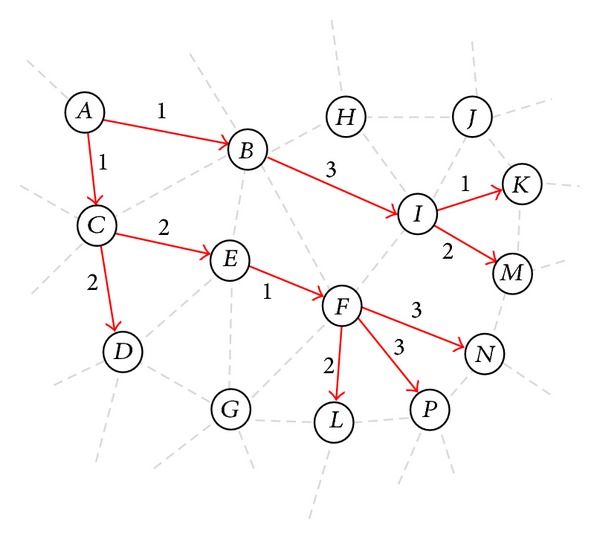
A typical multicast routing tree.

Since more transmissions take longer time on scheduling frame, minimizing the number of transmissions helps to improve the network's throughput. In a typical multicast/broadcast tree *T* ^*j*^, there are three kinds of nodes: source node *s* ^*j*^, forwarding nodes set (FWD^*j*^), and leaf nodes set (LF^*j*^). For example, consider the multicast tree shown in Figure 1. Here, the number associated with each link represents the channel assigned to that link. All nodes of tree, except the source node, have one parent. The source node (e.g., node *A*), as the root of the tree, sends data toward its children. A forwarding node (e.g., nodes *B*, *C*, *E*, *F*, and *I*) acts as both parent and child node; as a child node, it receives data from its parent, while in the role of a parent node, it sends the traffic toward its children. A leaf node (e.g., nodes *D*, *K*, *L*, *M*, *N*, and *P*) only plays the role of a child and receives data from its parent. It is clear from Figure 1 that NT_*A*_^*j*^ = NT_*B*_^*j*^ = NT_*C*_^*j*^ = NT_*E*_^*j*^ = 1, while NT_*I*_^*j*^ = NT_*F*_^*j*^ = 2 (i.e., NT(*T* ^*j*^) = 8).

Since we assume the bandwidth-guaranteed trees with bandwidth requirement tr_*s*_^*j*^, the created load by the *j*th session on the *i*th node can be generally formulated by the role of node and the number of its transmissions as

(5) $$\begin{array}{c} l_{i}^{j}=\{\begin{array}{cc} \text{t}{\text{r}}_{s}^{j}\left(1+\text{N}{\text{T}}_{i}^{j}\right) & \text{if}\;i\in \text{FW}{\text{D}}^{j},\\ \text{t}{\text{r}}_{s}^{j}\times \text{N}{\text{T}}_{i}^{j} & \text{if}\;i=s^{j},\\ \text{t}{\text{r}}_{s}^{j} & \text{if}\;i\in \text{L}{\text{F}}^{j},\\ 0 & \text{if}\;i\notin T^{j}. \end{array} \end{array}$$

Here, we define the utilization of the *i*th mesh router, denoted by *U*(*i*), as follows:

(6) $$\begin{array}{c} U\left(i\right)=\frac{l\left(i\right)}{C\left(i\right)}=\frac{1}{C\left(i\right)}\sum_{j=1}^{n_{s}}l_{i}^{j}, \end{array}$$

where *U*(*i*) indicates the percentage of the *i*th node's capacity used for routing of *n*_*s*_ multicast/broadcast sessions. For this case, the average utilization of the nodes is defined as

(7) $$\begin{array}{c} \bar{U}=\frac{1}{n}\sum_{i=1}^{n}U\left(i\right), \end{array}$$

where *n* is the total number of nodes in the network.

On the other hand, due to the shared nature of the wireless medium, adjacent transmissions cannot occur simultaneously on the same channel. To formulate this issue, we use the channel utilization concept defined in [17] with minor modifications. For the described MCMR-WMN model, consider a fixed transmission rate of *c*_0_. Each MAC multicast transmission in the *j*th routing tree uses a time fraction of the scheduling frame that is equal to tr_*s*_^*j*^/*c*_0_  . By definition, the utilization of channel *k* observed by node *y* (*X*_*y*_^*k*^) is the sum of the time fractions assigned to all nodes within the interference range of node *y* that are intended to transmit on channel *k*. Thus, considering *n*_*s*_ admitted multicast/broadcast sessions, the utilization of channel *k* observed by node *y* is formulated as

(8) $$\begin{array}{c} X_{y}^{k}=\sum_{j=1}^{n_{s}}\;\;\sum_{i\in \text{intf}(y)}\frac{\text{t}{\text{r}}_{s}^{j}}{c_{0}}q_{i,k}^{j}, \end{array}$$

where intf(*y*) denotes the set of interfering nodes located within the interference range of node *y*. For this case, the channel capacity constraint is given by

(9) $$\begin{array}{c} X_{y}^{k}\leq 1,\;\forall y\in V,\;\;\forall k\in \text{ch}\_\text{list}\left(y\right), \end{array}$$

where ch_list(*y*) indicates the set of assigned channels to the radios of node *y*. Since the radios of each node are assigned to different non-overlapping channels and no channel switching is allowed, one can show that condition (9) satisfies the described condition in (1). Therefore, the bandwidth-guaranteed multicast/broadcast sessions are feasible and schedulable if all interfering transmissions have a total load less than the normalized channel capacity. Different from the best-effort routing algorithms, quality of service (QoS) routing algorithms must use call admission control mechanisms to protect the QoS requirements of the existing flows [17, 21]. Clearly, it is desired to maximize the number of admitted sessions. In this regard, we define the network's throughput, denoted by *τ*, as the sum of the traffic load of all admitted feasible sessions as *τ* = ∑_*j*=1_^*n*_*s*_^tr_*s*_^*j*^.

## 5. Network's Throughput versus Resource Utilizations in MCMR-WMNs 

 In this section, we aim to derive analytical relationships for the network's throughput in terms of the node utilizations, the channel utilizations, and the number of transmissions.

**Theorem 2**

If all sessions have the same traffic load, for example, *tr*_*s*_^*j*^ = *T*_0_, and the capacity of all nodes in the network is identical, for example, *R*_*i*_ = *R* and *C*(*i*) = *C*, the average number of transmissions for the multicast flows is expressed as

(10) $$\begin{array}{c} \bar{NT}=\frac{nC}{n_{s}T_{0}}\bar{U}-\bar{W}, \end{array}$$

where *n*_*s*_ and $\bar{U}$ denote the number of admitted sessions and the average node utilization, respectively. In addition, $\bar{W}=\left(1/n_{s}\right)\sum_{j=1}^{n_{s}}W^{j}$ shows the average number of links in the tree of each session and *W* ^*j*^ is the number of links in the *jth* multicast tree.

**Proof**

Under the assumption *C*(*i*) = *C* and using (6) and (7), we have

(11) U¯=1nC∑j=1ns ∑i=1nlij=(a)1nC∑j=1ns(∑i∈FWDjtrsj(1+NTij)+trsjNTsj+∑i∈LFjtrsj)=T0nC∑j=1ns(∑i∈{sj,FWDj}NTij+|FWDj|+|LFj|),

where (*a*) comes from (5) and the fact that each multicast routing tree includes three kinds of nodes: a source node, forwarding nodes, and leaf nodes. In the above equations, NT_*s*_^*j*^ is the number of transmissions of source node, and |FWD^*j*^| and |LF^*j*^| denote the number of forwarding nodes and leaf nodes at the *j*th tree, respectively. On the other hand, from the graph theory [22]

(12) $$\begin{array}{c} \left|\text{FW}{\text{D}}^{j}\right|+\left|\text{L}{\text{F}}^{j}\right|=W^{j}. \end{array}$$

Thus, (11) can be simplified as

(13) U¯=T0nC∑j=1ns(∑i∈{sj,FWDj}NTij+Wj)=T0nC[∑j=1ns  ∑i∈{sj,FWDj}NTij+∑j=1nsWj].

According to (3) and (4) and considering the fact that NT_*i*_^*j*^ = 0 for *i* ∉ {*s* ^*j*^, FWD^*j*^}, we have

(14) $$\begin{array}{c} \bar{\text{NT}}=\frac{1}{n_{s}}\sum_{j=1}^{n_{s}}\;\;\sum_{i\in \{s^{j},\text{FW}{\text{D}}^{j}\}}\text{N}{\text{T}}_{i}^{j}. \end{array}$$

Thus,

(15) $$\begin{array}{c} \bar{U}=\frac{T_{0}}{nC}\left[n_{s}\bar{\text{NT}}+n_{s}\bar{W}\right]=\frac{n_{s}T_{0}}{nC}\left[\bar{\text{NT}}+\bar{W}\right]. \end{array}$$

As a result, $\bar{\text{NT}}=\left(nC/n_{s}T_{0}\right)\bar{U}-\bar{W}$.

**Corollary 3**

For the broadcast case, (12) can be rewritten as |*FWD* ^*j*^ | +|*LF* ^*j*^ | = *n* − 1 [22]. Thus,

(16) $$\begin{array}{c} \bar{NT}=\frac{nC}{n_{s}T_{0}}\bar{U}-n+1. \end{array}$$

In the rest of the section, we first present the problem for the small-scale MCMR-WMNs, and then, we extend our work to the case of large-scale MCMR-WMNs. In addition, due to the similarity of equations for the multicast and the broadcast sessions, we follow the problem only for the broadcast sessions.

*Small-Scale MCMR-WMNs.* In a small-scale MCMR-WMN, we suppose that all nodes are located in the interference range of each other. Thus, the channel utilization observed by any node is identical. For a small-scale MCMR-WMN, we define the utilization of channel *k* denoted by *X* ^*k*^ and the average channel utilization ${\bar{X}}_{\text{SS}}$ as follows:

(17) $$\begin{array}{c} X^{k}=\sum_{j=1}^{n_{s}}‍\;\;\sum_{i\in V}\frac{{\mathrm{tr}}_{s}^{j}}{c_{0}}q_{i,k}^{j},\;\forall k\in K, \end{array}$$

(18) $$\begin{array}{c} {\bar{X}}_{\text{SS}}=\frac{1}{K}\sum_{k=1}^{K}X^{k}. \end{array}$$

**Lemma 4**

Under the same conditions as in Theorem 2, the broadcast throughput of a small-scale MCMR-WMN is expressed in terms of the average node utilization and the average channel utilization, as follows:

(19) $$\begin{array}{c} \tau =\frac{c_{0}}{n-1}\left(nR\bar{U}-K{\bar{X}}_{SS}\right). \end{array}$$

**Proof**

Using (17) and averaging the utilization on different channels, we have

(20) $$\begin{array}{c} {\bar{X}}_{\text{SS}}=\frac{1}{K}\sum_{j=1}^{n_{s}}\;\;\sum_{k=1}^{K}\;\;\sum_{i\in V}\frac{\text{t}{\text{r}}_{s}^{j}}{c_{0}}q_{i,k}^{j}\overset{\left(a\right)}{=}\frac{n_{s}T_{0}}{Kc_{0}}\bar{\text{NT}}, \end{array}$$

where (*a*) comes from (2)–(4), and the assumption tr_*s*_^*j*^ = *T*_0_. Considering *τ* = *n*_*s*_ *T*_0_, *C* = *Rc*_0_, and replacing $\bar{\text{NT}}$ with the result in (16) for broadcast sessions, the throughput *τ* can be expressed as $\tau =\left(c_{0}/n-1\right)(nR\bar{U}-K{\bar{X}}_{\text{SS}})$.

*Large-Scale MCMR-WMNs.* Now, we extend the result of Lemma 4 to the large-scale MCMR-WMN case. In general, the channel utilization is a location-dependent parameter. However, due to the shared nature of the wireless medium, the channel utilizations observed by neighboring nodes are close to each other. Thus, considering the channel utilization observed by all nodes gives a lot of redundancy. One idea is to study the channel utilization observed by a special node on behalf of its neighbors. To address this solution, we define the “*interference domain* (*ID*)” and the “*interference domain head*” as follows.

**Definition 5**

The “*interference domain*" is defined as a subset of the network's nodes which satisfies three conditions.

- The interference domains have no common node that is, ID_*i*_⋂ID_*j*_ = *Ø*, *i* ≠ *j*.
- The interference domains span all nodes in the network that is, ⋃_*m*=1_^*M*^ID_*m*_ = **V**, where *M* denotes the total number of interference domains.
- Each interference domain, for example, the *m*th interference domain, includes a node denoted by *ξ*_*m*_ so that only the nodes of ID_*m*_ are located within the interference range of *ξ*_*m*_. We define *ξ*_*m*_ as the “*interference domain head*” of ID_*m*_.

**Figure 2:**
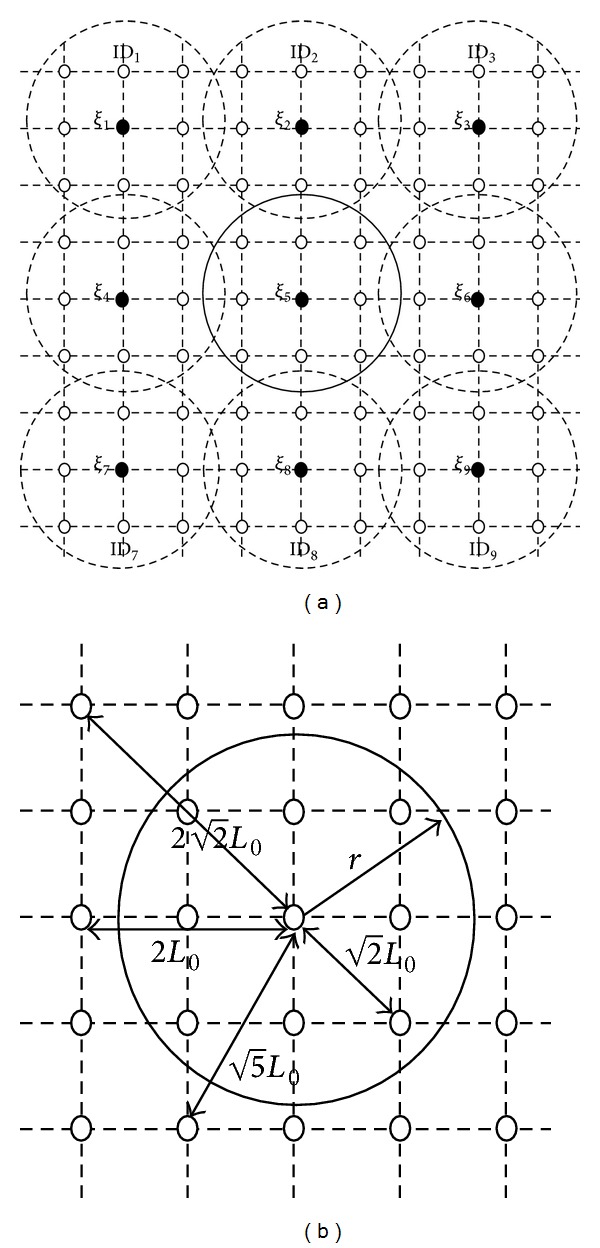
(a) A typical grid MCMR-WMN with its interference domains, (b) an interference domain.

It is clear from Definition 5 that a small-scale MCMR-WMN is a special case which consists of only one interference domain. The feasibility of condition (iii) is justified by the fact that mesh routers are usually deployed with careful planning. To clarify the above definition, consider a typical grid topology plotted in Figure 2 as a popular topology for the WMNs. Let the grid length be set to *L*_0_. In this case, for the interference range *d*_intf_, assume $\sqrt{2}L_{0}<d_{\text{intf}}<2L_{0}$ which is a reasonable interference range [2]. Thus, we can model the interference domains as a 3 × 3 square grids as shown in Figure 2(b). This modeling satisfies conditions (i)–(iii) in Definition 5. In Figure 2(a), each circle represents an interference domain and the central black nodes play the role of the corresponding interference domain head.

Now, let the network be composed of *M* interference domains ID_1_,…, ID_*M*_. For large-scale MCMR-WMNs, we define the average channel utilization ${\bar{X}}_{\text{LS}}$ as follows:

(21) $$\begin{array}{c} {\bar{X}}_{\text{LS}}=\frac{1}{MK}\sum_{k=1}^{K}\;\;\sum_{m=1}^{M}X_{m}^{k}, \end{array}$$

where *X*_*m*_^*k*^ denotes the utilization of channel *k* observed by the *m*th interference domain head.

**Theorem 6**

Assume all sessions have the same traffic load, for example, *tr*_*s*_^*j*^ = *T*_0_. The network's throughput of the large-scale MCMR-WMN is obtained as

(22) $$\begin{array}{c} \tau =\frac{MKc_{0}}{\bar{NT}}{\bar{X}}_{LS}. \end{array}$$

**Proof**

According to (8) and considering the condition (iii) in Definition 5, the utilization of the channel *k* observed by the *m*th interference domain head is given by

(23) $$\begin{array}{c} X_{m}^{k}=\sum_{j=1}^{n_{s}}\;\;\sum_{i\in \text{I}{\text{D}}_{m}}\frac{\text{t}{\text{r}}_{s}^{j}}{c_{0}}q_{i,k}^{j},\;\forall k\in K,\;\;m=1,\ldots ,M. \end{array}$$

Using (23) and averaging the utilization on different channels and different interference domain heads, we have

(24) $$\begin{array}{c} {\bar{X}}_{\text{LS}}=\frac{1}{MK}\sum_{j=1}^{n_{s}}\;\;\sum_{m=1}^{M}\;\;\sum_{i\in \text{I}{\text{D}}_{m}}\;\;\sum_{k=1}^{K}\frac{\text{t}{\text{r}}_{s}^{j}}{c_{0}}q_{i,k}^{j}, \end{array}$$

(25) $$\begin{array}{c} {\bar{X}}_{\text{LS}}\overset{\left(a\right)}{=}\frac{T_{0}}{MKc_{0}}\sum_{j=1}^{n_{s}}\;\;\sum_{m=1}^{M}\;\;\sum_{i\in \text{I}{\text{D}}_{m}}\;\;\sum_{k=1}^{K}q_{i,k}^{j}, \end{array}$$

where (*a*) comes from assumption tr_*s*_^*j*^ = *T*_0_. Under conditions (i) and (ii) in Definition 5 and using (2)–(4), we obtain

(26) $$\begin{array}{c} \bar{\text{NT}}=\frac{1}{n_{s}}\sum_{j=1}^{n_{s}}\sum_{m=1}^{M}\sum_{i\in \text{I}{\text{D}}_{m}}\sum_{k=1}^{K}q_{i,k}^{j}. \end{array}$$

Thus, (25) can be simplified as

(27) $$\begin{array}{c} {\bar{X}}_{\text{LS}}=\frac{n_{s}T_{0}\bar{\text{NT}}}{MKc_{0}}. \end{array}$$

As a result, since *τ* = *n*_*s*_ *T*_0_, the network's throughput can be obtained as $\tau =\left(MKc_{0}/\overset{-}{\text{NT}}\right){\bar{X}}_{\text{LS}}$.

**Corollary 7**

Under the same conditions as in Theorem 2, considering *C* = *Rc*_0_ and replacing $\bar{NT}$ with the result in (16), the broadcast throughput of a large-scale MCMR-WMN is expressed in terms of the average node utilization and the average channel utilization as follows:

(28) $$\begin{array}{c} \tau =\frac{c_{0}}{n-1}\left(nR\bar{U}-MK{\bar{X}}_{LS}\right). \end{array}$$

It is clear that different parameters of the network interact with each other. Thus, it is not possible to draw a specified boundary between them. Due to the limited number of radios and non-overlapping channels, proper use of the resources could improve the performance of the network. In this regard, as we will show in the next section, the number of transmissions and the load-balancing significantly affect the network's throughput.

## 6. Numerical Results

**Figure 3:**
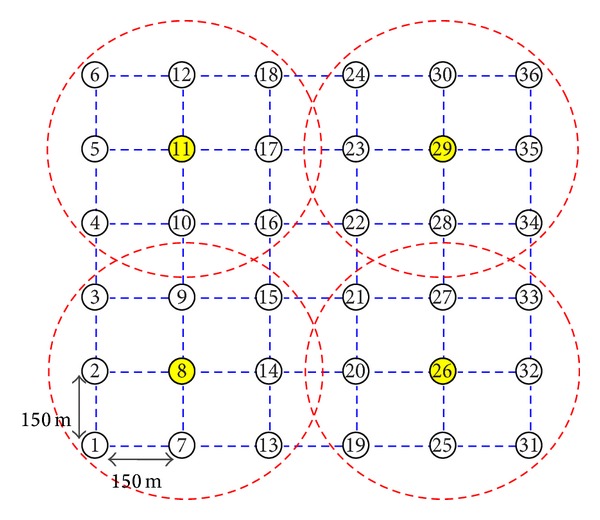
The grid topology considered in the simulations.

 In this section, we present a comprehensive evaluation on the relationship between the network's throughput and the resource utilizations. For this purpose, we apply the following protocols in a single-rate framework: SPT-JCRS [7], MCM-JCRS [7], IRMT [20], and IRBT [20]. In our Matlab simulation setup, as shown in Figure 3, we consider a 6 × 6 square grid with *n* = 36 and *M* = 4, where nodes 8, 11, 26, and 29 are the interference domain heads. The grid length (the distance between neighbor nodes in the same row or column) and the interference range are set to 150 m and 280 m, respectively. We also use the random channel assignment, in which the radios of each node are randomly assigned to the distinct channels. Obviously, in the cases that the number of channels is less than or equal to the number of radios, this method will act as the common channel assignment strategy.

**Figure 4:**
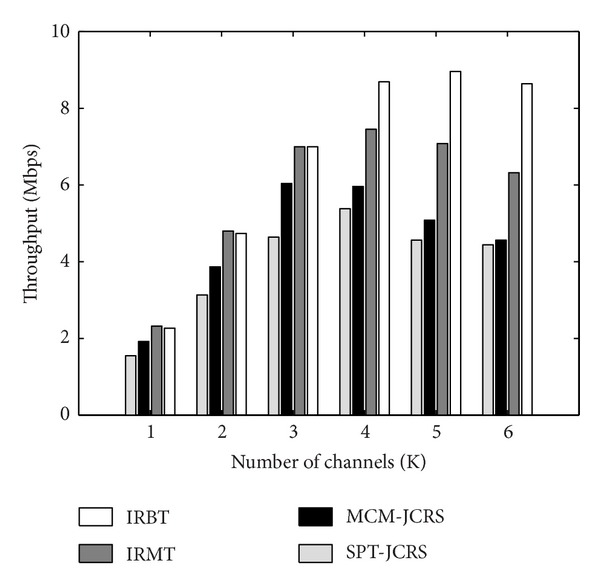
Network's throughput as function of the number of channels.

 In the simulations, the broadcast session requests arrive one by one at the network without any knowledge of the future requests. The source of each session is selected randomly. In addition, the traffic model of all sessions is assumed to be Constant Bit Rate (CBR) with tr_*s*_^*j*^ = 0.4 Mbps. Assuming *R* = 3, *c*_0_ = 12 Mbps, and 25 broadcast session requests, we study the performance of the network for different number of channels; that is, *K* = 1,…, 6. It is clear that in the case of *K* = 1, we have a SCSR-WMN.  Figure 4 compares the throughput of the aforementioned protocols in terms of the number of channels *K*. In addition, Table 1 shows the simulation results in more details. It should be noted that each data point is obtained by averaging the results of 15 individual runs on different randomly experiments. In this table, $\bar{\text{NT}}$, $\bar{U}$, ${\bar{X}}_{\text{LS}}$, and *τ*_sim_ present the experimental results obtained for the average number of transmissions, the average node utilization, the average channel utilization, and the network's throughput, respectively. It is worth noting that the results in Table 1 exactly follow the described theoretical relationships in (16), (22), and (28). As an example, Table 1 compares *τ*_sim_ with the theoretical throughput *τ*_theory_ extracted from (28). Obviously, *τ*_sim_ is similar to *τ*_theory_ for different number of channels. This shows the validity of our analysis. This comparison can be also verified for relationships (16) and (22). It is clear that different parameters of the network interact with each other. In this situation, given the limited number of radios and channels, proper use of the resources could improve the performance of the network. Actually, using an efficient traffic engineering mechanism leads to better spectrum utilization and increases the fairness in the network. Thus, more resources will be available for accepting the future sessions and the overall throughput will be increased. In this regard, it is observed that the performance of the IRBT and the IRMT algorithms much better than that of the other two algorithms. In fact, the IRBT and the IRMT algorithms jointly address the transmission channel selection and the load-balanced routing tree construction [20]. These schemes not only take into account the number of transmissions, but also consider both inter-flow and intra-flow interferences to route the sessions through alternative feasible paths. Thus, the traffic load is balanced in the network. However, the MCM-JCRS and the SPT-JCRS algorithms cannot efficiently use the resources of the network due to being limited to non-interference-aware routing trees.

In [20], we demonstrated that the IRBT algorithm balances the traffic load in the network more efficiently than the IRMT algorithm. The results in Table 1 also confirm this issue. From this table, we can see that the IRBT approach improves the utilization of the network resources. For *K* = 1,2, 3 (i.e., common channel assignment), although the IRMT algorithm leads to less number of transmissions than the IRBT algorithm, the load-balancing ability of the IRBT makes both schemes have the same network's throughput. If the traffic load in the network is balanced, the interference will be decreased, and consequently, the call acceptance rate will be increased. In contrast, for *K* > 3, both IRMT and IRBT algorithms nearly have the same number of transmissions. In this situation, the load-balancing factor plays more efficient role in the network's performance. This causes the IRBT algorithm to show better throughput.

On the other hand, by increasing the number of channels, first the network's throughput linearly increases. However, for *K* > 3, it is gradually saturated. Due to the random channel assignment strategy, further increasing of the channels leads to the less number of common channels between the neighbor nodes. Thus, the possibility of enjoying the wireless broadcast advantage will be decreased. This increases the number of transmissions as shown in Table 1. In this situation, the lack of load-balancing could sufficiently reduce the network's throughput.

## 7. Conclusion

 In this paper, the throughput of a MCMR-WMN was quantified. We focused on the scenario of on-demand QoS multicast/broadcast sessions, where each session has a specific bandwidth requirement. In particular, considering the resource constraints, we derived analytical relationships for the network's throughput in terms of the node utilization, the channel utilization, and the number of transmissions. This gives simple solutions for the future designs to predict the network's throughput based on the resource utilizations. In line with the proposed relationships, we also demonstrated that the network's throughput is significantly affected by both number of transmissions and degree of load-balancing. On one hand, minimizing the number of transmissions reduces the use of the network resources. On the other hand, load-balancing increases the fairness in the network. In this situation, more resources will be available for accepting the future sessions. Thus, the overall network's throughput will be increased.

## Figures and Tables

**Table 1:** Performance comparison for different number of channels.

Number of channels	Algorithm	$\overset{-}{\text{NT}}$	$\overset{-}{U}$	${\overset{-}{X}}_{\text{LS}}$	*τ*_sim_ (M bps)	*τ*_theory_ (M bps)
*K* = 1	IRBT	20.002	0.0961	0.9428	2.2667	2.2655
	IRMT	15.9444	0.0912	0.77	2.32	2.321
	MCM-JCRS	18.1	0.0787	0.7239	1.92	1.9214
	SPT-JCRS	23.95	0.0703	0.7717	1.5467	1.5448

*K* = 2	IRBT	19.7273	0.1998	0.9722	4.7333	4.7317
	IRMT	15.9967	0.1888	0.7993	4.8	4.7986
	MCM-JCRS	17.8833	0.1578	0.7205	3.8667	3.8669
	SPT-JCRS	23.3780	0.1411	0.7628	3.1333	3.1325

*K* = 3	IRBT	20.0016	0.2970	0.9719	7	6.9988
	IRMT	16.2761	0.2769	0.7911	7	6.9984
	MCM-JCRS	18.225	0.2481	0.7644	6.04	6.0418
	SPT-JCRS	23.4697	0.2093	0.7561	4.64	4.6392

*K* = 4	IRBT	20.2207	0.3702	0.9144	8.6909	8.6918
	IRMT	18.6563	0.3086	0.7244	7.4545	7.4531
	MCM-JCRS	18.966	0.2483	0.589	5.9636	5.9631
	SPT-JCRS	24.1418	0.2455	0.6761	5.3818	5.3816

*K* = 5	IRBT	21.5448	0.3909	0.8040	8.96	8.9613
	IRMT	20.6932	0.3043	0.6105	7.08	7.0815
	MCM-JCRS	21.7823	0.2225	0.4608	5.08	5.0791
	SPT-JCRS	26.238	0.2155	0.4987	4.56	4.56

*K* = 6	IRBT	23.9454	0.3929	0.7182	8.64	8.6388
	IRMT	23.6876	0.2862	0.5197	6.32	6.3212
	MCM-JCRS	24.2579	0.2088	0.3854	4.56	4.5603
	SPT-JCRS	27.2301	0.2133	0.4204	4.44	4.4389
